# Fibrous Dysplasia of Parietal Bone: A Case Report and Review of Literature

**DOI:** 10.7759/cureus.73023

**Published:** 2024-11-04

**Authors:** Shuruq Albushi, Maryam Almohalwas, Fatimah Kushk, Rawan Alhejaili, Razan Samman

**Affiliations:** 1 Department of Neurosurgery, King Fahad General Hospital, Medina, SAU; 2 Department of Neurosurgery, Taibah University, Medina, SAU; 3 Department of General Medicine, King Salman Bin Abdulaziz Medical City, Medina, SAU; 4 Department of General Medicine, Miqat General Hospital, Medina, SAU; 5 Department of Internal Medicine, Prince Mohammed Bin Abdulaziz Hospital, Medina, SAU

**Keywords:** benign tumour, bone disorder, fibrous dysplasia, mutation, parietal bone

## Abstract

Fibrous dysplasia (FD) is a rare, benign, and slowly progressive bone disorder that affects one or more bones, where the normal bone is replaced by atypical fibrous connective tissue, making the bone weak, fragile, and more susceptible to fracture. FD can affect a single bone (monostotic FD) or multiple bones (polyostotic FD). The clinical manifestations and progression of FD vary from one individual to the other. The diagnosis is based on radiological and histopathological findings; a bone biopsy is the diagnostic test of choice for FD. The management of FD depends on the type and severity of the condition. The modality of management is mainly conservative. However, surgery is offered to preserve the function and prevent complications. Surgery is the ideal treatment that helps in definitive diagnosis, determining disease progression or malignant transformation, removing a compressive lesion, cosmetic surgeries, and failure of non-surgical treatments. We reported a new case of monostotic fibrous dysplasia involving the right partial bone in a 27-year-old male who underwent a successful operative excision of the lesion. The patient clinically improved and was discharged later with instructions for his follow-up. We report this case of monostotic FD as it highlights the importance of considering FD in the differential diagnosis of bone lesions, especially in young patients presenting with bone pain or deformities.

## Introduction

Fibrous dysplasia (FD) is a benign (non-neoplastic) developmental disorder of one or more bones, presenting as a slowly progressive replacement of normal bone by abnormal fibrous connective tissue [1,2]. Researchers believe that FD is caused by incorrect differentiation of osteoblasts due to a mutation in the GNAS gene that leads to the replacement of normal bone marrow by woven bone and fibrous tissue [1,3]. FD is a rare non-familial disease representing almost 2.5% of all bone lesions and 7% of all benign bone tumors [1]. Male to female ratio is about 2:1 and is seen more commonly in the first two decades of life [4]. Three clinical types have been described: involvement of single bone (known as monostotic form - MFD) accounting for 70% of cases, multiple bones (known as polyostotic form - PFD) accounting for 30% of cases, and McCune-Albright Syndrome (MAS) which is a triad of PFD, precocious puberty, and café-au-lait skin maculae [1]. Craniofacial manifestations occur in 30% of the patients with MFD and 50% of patients with PFD [2].

FD typically manifests as a gradual, painless enlargement of the affected bones, usually in the craniofacial region, leading to noticeable facial asymmetry [1]. The severity of the disorder varies from one individual to another; some patients may describe a dull, aching pain that is aggravated by activity and improved by rest [3,4]. Patients with temporal bone involvement typically present with conductive hearing loss, temporal mass, and external auditory canal stenosis [5]. Diagnosis is primarily based on radiological and histopathological findings; bone biopsy is the diagnostic test of choice of FD. Additional non-invasive imaging studies such as X-rays, CT, MRI, and bone scans can provide further evaluation of the lesion. On imaging, FD typically appears with a "ground glass" appearance with central dense sclerotic and radiolucent fibrotic regions [6]. The modality of treatment is mainly conservative. However, surgery must be considered to preserve function and prevent complications [4].

## Case presentation

**Figure 1:**
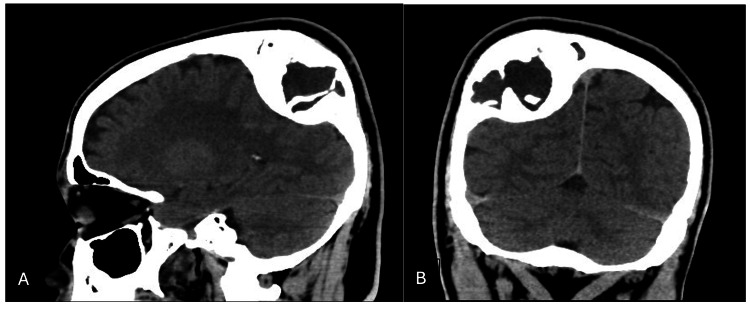
Preoperative non-contrast computed tomography scan. Both sagittal (A) and coronal (B) planes show hypertrophied right parietal sclerotic bone lesions, compatible with fibrous dysplasia.

A 27-year-old Burundian male has been medically free and presented with painful swelling in the right parietal region for the last 20 years. The swelling gradually enlarged through the years; the pain was dull, aching in nature, intermittent in course, with no radiation, aggravated by applying pressure on it, 6/10 in severity. There was no history of fever, weight loss, anorexia, night sweats, nausea, vomiting, headache, loss of consciousness, papilledema, seizures, or focal neurological deficit. Local examination showed there was a scalp swelling of 5×5×6 cm at the right parietal region, normal temperature, non-compressible, nor pulsatile, hard in consistency with no skin discoloration. His neurological examination was normal. The computerized tomographic (CT) imaging without contrast revealed a large intra-osseous mixed cystic and sclerotic lesion at the right high parietal bone measuring 6×5×7 cm (Figure 1).

**Figure 2:**
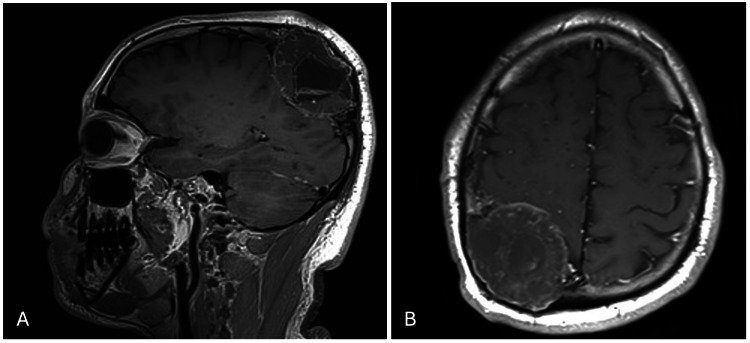
Preoperative contrasted T1 weighted image magnetic resonance imaging (MRI). Both sagittal (A) and axial (B) planes show right parietal heterogeneously enhancing lesion with an internal cystic component.

Magnetic resonance imaging (MRI) of the brain showed a benign-looking right parietal diploic space with a large heterogeneously enhancing lesion in the internal area with a cystic component measuring 6.4×5.5×5.5 cm AP (anteroposterior) × TS (transverse) × CC (craniocaudal). No periosteal reaction or intraparenchymal invasion, infarction, hemorrhage, space-occupying lesion, edema, mass effect, midline shift, hydrocephalus, with posterior fossa, orbit, paranasal sinuses, and mastoid cells structures unremarkable (Figure 2). 

**Figure 3:**
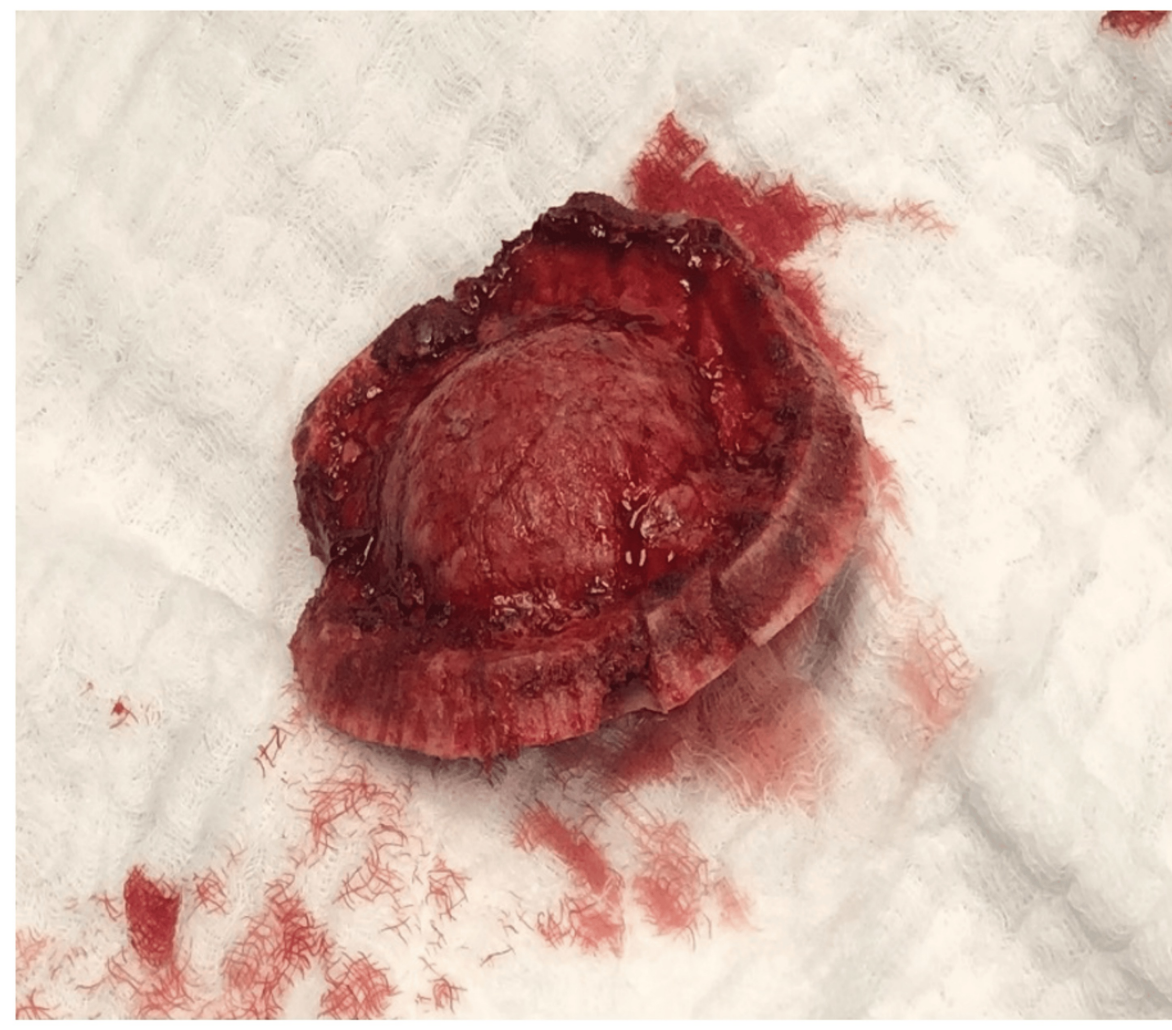
The bony piece sample measuring 10×9×4.5 cm.

**Figure 4:**
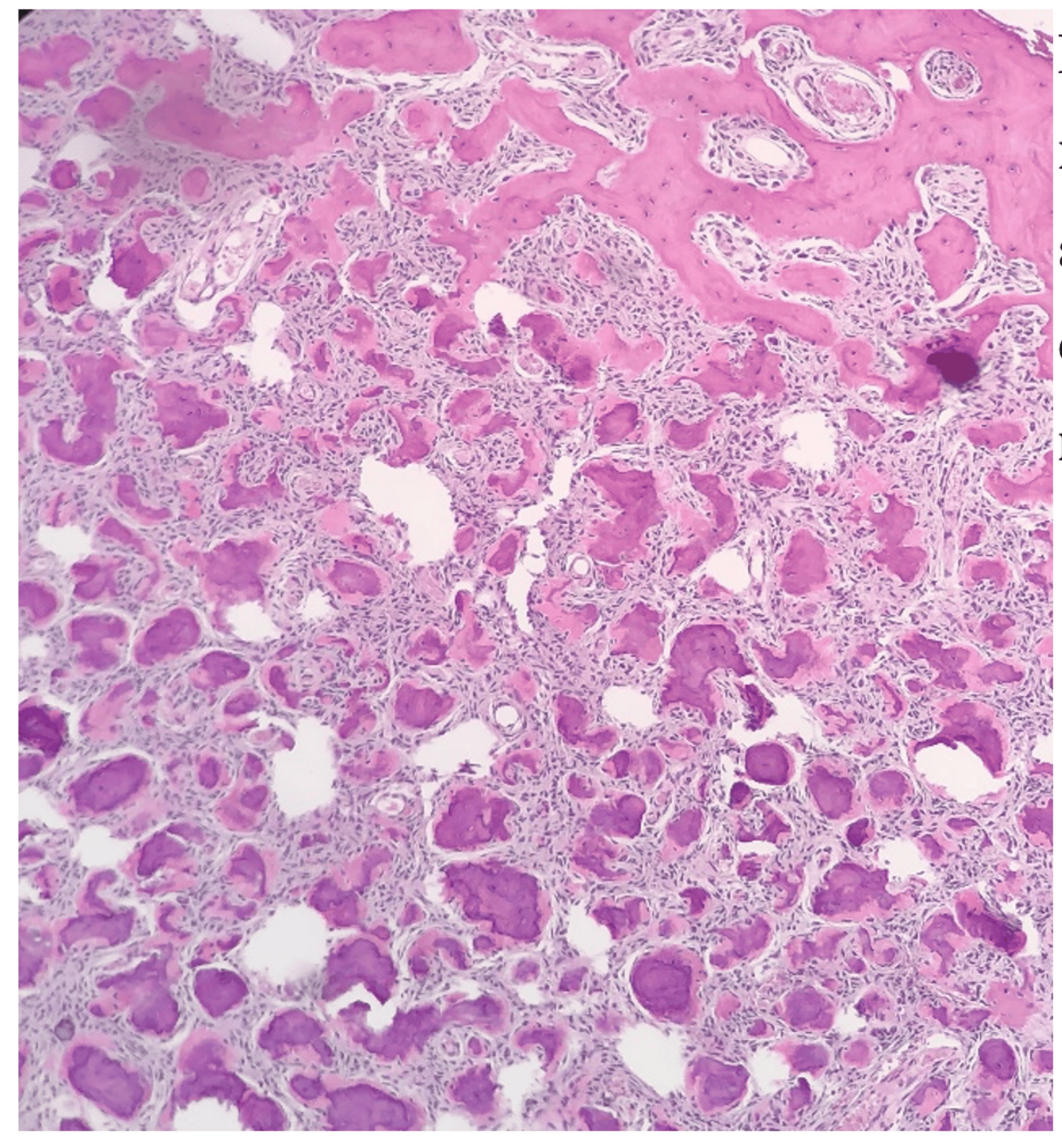
The pathology section revealed trabeculae of woven bone and intervening fibrous stroma containing bland spindle cells with no cytological atypia.

Subsequently, he underwent surgery for the removal of the mass and histopathological confirmation. The sample was received as a bony piece measuring 10×9×4.5 cm (Figure 3). The cortex is intact and contains a tan-white tumor underneath. The pathology section revealed trabeculae of woven bone without osteoblastic rimming and intervening fibrous stroma containing bland spindle cells with no cytological atypia (Figure 4). Based on the biopsy, the diagnosis of FD was confirmed.

The patient had an uneventful postoperative recovery. A drain placed during the surgery was removed on the 3rd postoperative day (POD), and the patient continued to receive antibiotics until the 7th POD. He was discharged home on the 10th POD, with a follow-up appointment scheduled in one week. Instructions were provided for wound care and dressing changes every other day until the sutures were removed on the 14th POD. The wound healed well. Regular follow-ups with physical examination, MRI, and CT imaging were provided. The patient remained symptom-free during the six-month follow-up after the surgical excision without recurrence noted on imaging.

## Discussion

FD is a rare benign bone disorder that affects the growth and development of bones. In this condition, a portion of the bone is replaced by fibrous connective tissue and poorly formed trabecular bone, originating in the medullary cavity. This condition is thought to result from a postzygotic mutation in the guanine nucleotide stimulatory protein (GNAS1) gene, which is responsible for producing a protein called Gsα. This protein plays a key role in regulating bone growth and development. The mutation in the GNAS gene causes the Gsα protein to become overactive, leading to abnormal bone growth and development [7]. It affects people of all races, genders, and ages. It is not known to be hereditary, and there is no known family history associated with it. Epidemiological studies have shown that the prevalence of FD is similar across all racial and gender groups, and the average age of diagnosis is between 10 and 30 years old. This condition accounts for approximately 5-7% of benign bone tumors [8].

There are three main types of FD. Monostotic FD is the most common type of FD, which is characterized by the presence of a single bone lesion. It accounts for approximately 75% of cases and usually affects 20-30-year-olds [1]. It most often involves the femur or ribs, while craniofacial bone involvement occurs in about 25% of cases. The mandible and maxilla are the most affected craniofacial bones, followed by occipital, parietal, and frontal bones [2]. On the other hand, polyostotic FD is characterized by the presence of multiple bone lesions. This condition typically affects long bones, ribs, and the skull, often occurring unilaterally. In cases of polyostotic FD, there is nearly 100% involvement of the craniofacial bones with the ethmoid, sphenoid, frontal, maxillary, temporal, and occipital bones affected in that order of frequency [9]. It accounts for approximately 30% of the lesion and usually affects people below 10 years old, and it is more common in females. The third type of FD is McCune-Albright syndrome (MCA), which occurs in 3-5% of cases and is characterized by a classic triad consisting of polyostotic FD lesions, cutaneous hyperpigmentation ("café au lait macules"), and endocrine abnormalities (precocious puberty, acromegaly, hyperthyroidism, Cushing’s syndrome) to which bone affected by FD is more sensitive to these hormonal changes than unaffected bone [10]. Patients with FD can present with a variety of neurological symptoms, including headaches, seizures, visual disturbances, and cognitive impairment. Headaches are often caused by increased intracranial pressure due to the presence of abnormal fibrous tissue. Seizures can be caused by abnormal electrical activity in the brain. Visual disturbances can be caused by the abnormal tissue pressing on the optic nerve, leading to blurred vision or even blindness. Cognitive impairment can be caused by the abnormal tissue pressing on the brain, leading to difficulty with memory, concentration, and other cognitive functions. In addition, patients may also experience muscle weakness, numbness, and tingling due to the abnormal tissue pressing on the nerves [11].

CT imaging is the preferred imaging modality for the evaluation of FD, they provide detailed images of the bone and can help to distinguish between FD and other bone disorders. The lesion appears as a lytic lesion found in the metaphysis or diaphysis, displaying a "ground glass" matrix. There may be an expansion of the bone and potential bowing. The cortical bone appears thinned, and endosteal scalloping is observed. Typically, there is no periosteal reaction unless a pathologic fracture occurs. MRI is generally unnecessary unless there are concerns about a radiographically occult pathologic stress reaction or fracture [12]. Management of FD depends on the type and severity of the condition. The medical management is usually based on bisphosphonates. Bisphosphonates can help reduce the size of the lesions caused by FD and can also help reduce pain and improve mobility. The most used bisphosphonate for FD is pamidronate. Studies have shown that pamidronate can reduce the size of FD lesions and improve bone density. Previous studies documented the role of steroids, which are used to reduce inflammation and pain and to reduce the size of the lesions caused by the disorder. Steroids can also be used to reduce the risk of fractures and to improve the overall quality of life for those affected by the disorder. Surgery is the best treatment option for a definitive diagnosis. It helps determine disease progression or malignant degeneration, removes compressive lesions for cosmetic purposes, and addresses failed nonsurgical therapy. The most common surgical treatment for FD is known as curettage and bone grafting. This procedure involves complete surgical resection of the affected bone, followed by reconstruction using bone grafts or materials like titanium plates and screws. If the affected area is too large or significant to be entirely removed, the surgeon may reduce the size of the bone to its normal dimensions using a high-speed burr [11]. Larger reconstruction surgeries involving bone graft placement have lower recurrence rates (45%) compared to less aggressive recontouring surgeries (82%) [13]. Surgical procedures should aim at restoring bone function and preventing complications related to the location of lesions on the skull while achieving a satisfactory cosmetic outcome. Radiation therapy is generally avoided as it can potentially induce malignant transformation. 

In our case, the patient presented with painful swelling in the right parietal region for the last 20 years. The patient underwent successful surgical removal of the mass, and the histopathology confirmed the diagnosis of monostotic FD of the parietal bone.

## Conclusions

Fibrous dysplasia (FD) is a rare benign bone disorder characterized by the gradual replacement of normal bone with fibro-osseous connective tissue. The abnormal fibrous tissue makes the bone weak, fragile, and more prone to fractures. In addition to pathological fractures, the formation of secondary aneurysmal bone cysts and, in rare cases, malignant changes are the main complications of FD. The monostotic FD is usually asymptomatic; however, in some cases, it can lead to pain, deformity, and pathological fractures, making early detection and management important for preventing complications and improving patient outcomes. We report this case of monostotic FD as it highlights the importance of considering FD in the differential diagnosis of bone lesions, especially in young patients presenting with bone pain or deformities.
